# Dataset for verification of a vibro-acoustic coupling model and optimized results on sectional geometries of two sealing rubber models

**DOI:** 10.1016/j.dib.2020.106038

**Published:** 2020-07-18

**Authors:** Guoming Deng, Jianwang Shao, Songlin Zheng, Xian Wu

**Affiliations:** School of Automotive Studies, Tongji University, Shanghai 201804, PR China

**Keywords:** Rubber layer, Vibro-acoustics, Sine-auxiliary function, Radiated sound power, Simulated annealing algorithm, Hybrid FE-SEA, Structural vibration, Acoustic insulation

## Abstract

This data article comprises collected data to verify a vibro-acoustic coupling model and raw records of optimized results on sectional geometries of two simplified sealing rubber models. The dataset is generated based on both analytical methods and numerical solutions, including Finite Element Simulation and Hybrid Finite Element - Statistic Energy Analysis Simulation. The calculations have been performed through a 2.3GHz PC with Intel Xeon Core E5-2658 v4 and 128 GB RAM in Tongji University.

All the results from these data will help researchers and engineers in vibro-acoustic coupling analysis of dual-membrane or combined dual-membrane models with a sine-auxiliary function and advanced understanding of the coupling characteristics. One of the main original contributions is also to share the data sets to give the opportunity to researchers for testing and validating numerical models of the vibro-acoustic coupling problem. In addition, the optimization characteristics of sectional geometries of the sealing rubber models based on a modified simulated annealing algorithm can retain further potential analysis with the data provided.

This Data in Brief article is an additional item directly alongside the following paper submitted in the Elsevier journal Applied Acoustics, “Optimal study on sectional geometry of rubber layers and cavities based on the vibro-acoustic coupling model with a sine-auxiliary function” [1], where the detailed interpretation of models and results can be found.

Specifications table

**Table:** 

Subject	Automotive Engineering
Specific subject area	Vibration and acoustics, mid-frequency and high-frequency noise, sealing noise
Type of data	ASCII files and *.mat files
How data were acquired	The data of analytical solutions, optimization study, and effects study are acquired based on written codes run in the commercial software MATLAB (Version R2018b). The data of FEM (Finite Element Method) verifications, including uncoupled and coupled models, are acquired from numerical simulations using commercial software Hypermesh (Version 13.0) and ABAQUS (Version 6.14-1). The data of sound transmission loss with Hybrid FE-SEA (Statistic Energy Analysis) simulation are obtained through the commercial software VA One (Version 2015).
Data format	Raw computational Analyzed
Parameters for data collection	The data collection was performed through a 2.3GHz PC with Intel Xeon Core E5-2658 v4 and 128GB RAM.
Description of data collection	Codes based on our proposed method and analytical methods were written for calculations. The FEM and Hybrid FE-SEA models were built through the aforementioned commercial software for verification and analysis. The data was collected from raw computational and analyzed results.
Data source location	Tongji University, No. 4800 Caoan Road, Shanghai 201804, PR China.
Data accessibility	With the article
Related research article	G.M. Deng, S.J. Wang, S.L. Zheng, X. Wu, Optimal study on sectional geometry of rubber layers and cavities based on the vibro-acoustic coupling model with a sine-auxiliary function, Applied Acoustics, in press, https://doi.org/10.1016/j.apacoust.2020.107522.

## Value of the data

• The computational and processed data will enable readers to access the detailed data in our work [1], and more importantly allow peer researchers to use these data as benchmarking to improve method or technique on a vibro-acoustic coupling model of combined dual-membrane types.
• Students, researchers, and engineers can take our data as benchmarking or the base to study other techniques and to further analyze vibro-acoustic coupling characteristics.
• The data of effects study on geometric parameters of sealing rubbers and cavities can be used for potential analysis on vibro-acoustic coupling mechanism among mid and high frequencies.
• The data of optimized results can be used for comparison with results of any other optimization algorithm.

## 1 Data description

The data presented in this article include two groups. One is the data for verification of a vibro-acoustic coupling model [2] using our proposed method. This group of data incorporates analytical and FEM solutions for modal frequencies of the vibro-acoustic coupling model. The first 100 modal frequencies for independent subsystems of the two panels and two cavities as well as their coupling system are involved in the group of data. Table 1 lists the description of data files in this group.

**Table 1:** List of data files in the first group in the dataset.

ID	File name	Description
1	Panel_a_Analytical.mat	Modal frequencies of the subsystem of “Panel a” obtained from written codes based on an analytical method.
2	Panel_a_FEM.txt	First 100 modal frequencies of “Panel a” obtained from the commercial software *Hypermesh/Optistruct.*
3	Panel_b_Analytical.mat	Modal frequencies of the subsystem of “Panel b” obtained from written codes based on an analytical method.
4	Panel_b_FEM.txt	First 100 modal frequencies of “Panel b” obtained from the commercial software *Hypermesh/Optistruct.*
5	Air_gap_Analytical.mat	Modal frequencies of the subsystem of “Air gap” obtained from written codes based on an analytical method.
6	Air_gap_FEM.txt	First 100 modal frequencies of “Air gap” obtained from the commercial software *ABAQUS.*
7	Enclosure_Analytical.mat	Modal frequencies of the subsystem of “Enclosure” obtained from written codes based on an analytical method.
8	Enclosure_FEM.txt	First 100 modal frequencies of “Enclosure” obtained from the commercial software *ABAQUS.*
9	Coupling_Analytical.mat	Modal frequencies of the vibro-acoustic coupling system obtained from written codes based on the proposed method and Du's method [3].
10	Coupling_Analytical.txt	First 100 modal frequencies of the coupling system obtained from the commercial software *ABAQUS*.
11	Modal_frequency_convergence.txt	The data for modal convergence of four natural frequencies of the coupling model.

The other group of data incorporates the results of optimization and effects study. The data for radiated sound power and effects’ results refer to frequency spectrums in the range of 800–4000 Hz with a resolution of approximately 4 Hz, which were acquired from MATLAB calculation based on written codes. The data for sound transmission loss of annulus models refer to frequency spectrums in 1/12 octave bands, acquired from the commercial software VA One. Table 2 lists the description of data files in this group.

**Table 2:** List of data files in the second group in the dataset.

ID	File name	Description
1	S1_Annulus_SoundTransmissionLoss.txt	Frequency spectrums of sound transmission loss of the S1 annulus model.
2	S1_RadiatedSoundPower.txt	Frequency spectrums of radiated sound power of the S1 vibro-acoustic coupling model*.*
3	S2_Annulus_SoundTransmissionLoss.txt	Frequency spectrums of sound transmission loss of the S2 annulus model.
4	S2_RadiatedSoundPower.txt	Frequency spectrums of radiated sound power of the S2 vibro-acoustic coupling model*.*
5	SA_algorithm_Process.txt	A process of the modified Simulated Annealing algorithm for the optimization of radiated sound power.
6	Effects_Lx.txt	Frequency spectrums of radiated sound power for effects study of the parameter Lx.
7	Effects_Lz.txt	Frequency spectrums of radiated sound power for effects study of the parameter Lz.
8	Effects_h1.txt	Frequency spectrums of radiated sound power for effects study of the parameter h1.
9	Effects_h2.txt	Frequency spectrums of radiated sound power for effects study of the parameter h2.

## 2 Experimental design, materials, and methods

The methodology to produce the data here presented is fully described in the article [1]. The following paragraphs briefly describe the computational protocols.

The written codes for calculating modal frequencies of structures are based on the analytical method for simply supported flexible panels which traditionally take the sine function as the admissible function. The written codes for calculating modal frequencies of cavities are based on the analytical method for rigid walls, which traditionally take the cosine function as the admissible function. The proposed method use cosine function added with a simple sine-auxiliary function as the admissible function for the cavities in dual-membrane or combined dual-membrane models. Codes can be written based on the proposed method. Material parameters, geometric parameters, boundary conditions, and model parameters are fully described in the article [1]. Consequently, by using the software MATLAB (Version R2018b), we can test the written codes and acquire analytical results. Verification can be performed based on the data.

FEM models corresponding to the same vibro-acoustic coupling model and its subsystems can be built in the environment of the commercial software Hypermesh (Version 13.0). The built model files for the structures are then executed by the solver Hypermesh/Optistruct (Version 13.0). The built model files for the cavities and the whole coupling model are executed by the solver ABAQUS/Standard (Version 6.14-1). Material parameters, geometric parameters, and boundary conditions correspond to those for analytical calculations. Detailed model parameters, including element size, element types and boundary addressing, are fully described in the article [1]. Thus, we can acquire the FEM results and take the verification based on the data.

The proposed method and a numerical method for evaluating radiated sound power [1] are based on to write codes so as to calculate the radiated sound power of both two vibro-acoustic coupling models. We have designed an integrated power as the objective function and used bounds of design variables according to real use in the automotive industry for optimization. A modified simulated annealing algorithm is proposed to do the optimization. The codes are carried out in MATLAB. After the optimization work, the optimized results with lower radiated sound power can be obtained.

Optimized cases are then selected to build Hybrid FE-SEA models based on the same geometric parameters, material parameters, and boundary conditions. But a model that more approaches a bulb seal, i.e., an annulus-section model is chosen to calculate the sound transmission loss through the commercial software VA One (Version 2015). Thus, the data of sound transmission loss can be acquired.

## Declaration of Competing Interest

The authors declare that they have no known competing financial interests or personal relationships which have, or could be perceived to have, influenced the work reported in this article.

## Appendix Supplementary materials

Image, application 1
